# MicroRNAs as Biomarkers and Therapeutic Targets for Acute Kidney Injury

**DOI:** 10.3390/diagnostics13182893

**Published:** 2023-09-09

**Authors:** Kenji Tsuji, Hiroyuki Nakanoh, Kazuhiko Fukushima, Shinji Kitamura, Jun Wada

**Affiliations:** 1 Department of Nephrology, Rheumatology, Endocrinology and Metabolism, Okayama University Graduate School of Medicine, Dentistry and Pharmaceutical Sciences, Okayama 700-8558, Japan; 2 Program in Membrane Biology, Center for Systems Biology, Department of Medicine, Division of Nephrology, Massachusetts General Hospital, Harvard Medical School, Boston, MA 02114, USA; 3 Department of Nursing Science, Faculty of Health and Welfare Science, Okayama Prefectural University, Okayama 719-1197, Japan

**Keywords:** microRNA, acute kidney injury, biomarker, mesenchymal stem cell

## Abstract

Acute kidney injury (AKI) is a clinical syndrome where a rapid decrease in kidney function and/or urine output is observed, which may result in the imbalance of water, electrolytes and acid base. It is associated with poor prognosis and prolonged hospitalization. Therefore, an early diagnosis and treatment to avoid the severe AKI stage are important. While several biomarkers, such as urinary L-FABP and NGAL, can be clinically useful, there is still no gold standard for the early detection of AKI and there are limited therapeutic options against AKI. miRNAs are non-coding and single-stranded RNAs that silence their target genes in the post-transcriptional process and are involved in a wide range of biological processes. Recent accumulated evidence has revealed that miRNAs may be potential biomarkers and therapeutic targets for AKI. In this review article, we summarize the current knowledge about miRNAs as promising biomarkers and potential therapeutic targets for AKI, as well as the challenges in their clinical use.

## 1. Introduction

Acute kidney injury (AKI) is a clinical syndrome where a rapid decrease in kidney function and/or urine output is observed, which may result in the imbalance of water, electrolytes, and acid base, due to a variety of causes, including sepsis, major surgery, hypovolemia, drug toxicity, urinary tract obstruction, and rhabdomyolysis [1]. It is reported that the prevalence of AKI is 23.2 % for inpatients [2] and 57.3% in intensive care unit (ICU) patients [3]. The mortality of AKI is 4.9% in all AKI, 3.4 % in stage 1, 7.5% in stage 2, 13.2% in stage 3, and 24.1% in dialysis-dependent patients [2]. AKI, which in outpatients is about 70% pre-renal and in inpatients is 55–60% intra-renal, is mostly induced either by acute tubular necrosis (ATN) caused by ischemia due to sepsis, or by NSAIDs, antibiotics, cisplatin or contrast agents [4]. In addition, anti-cancer agents in current development increase drug-induced AKI. For example, an immune checkpoint inhibitor may cause tubulointerstitial nephritis [5], and a vascular endothelial growth factor (VEGF) inhibitor may cause thrombotic microangiopathy (TMA) [6]. In addition, patients with AKI tend to require extended hospitalization, leading to a significant financial burden. Therefore, an early diagnosis and treatment to avoid the severe AKI stage would be important. For the purpose of an early diagnosis and the establishment of common diagnostic criteria, the concept of AKI was distinguished from acute renal failure (ARF) [7]. Subsequently, several criteria for classifying AKI were developed: risk, injury, failure, loss of kidney function, and end-stage kidney disease (RIFLE); the acute kidney injury network (AKIN); and the kidney disease improving global outcomes (KDIGO) [1]. In these guidelines, increases in serum creatinine (s-Cr) and/or decreases in urine output were the important criteria. An early diagnosis may not always be easy according to the guidelines, partly because s-Cr does not reflect direct tubular injury; thus, biomarkers over s-Cr and urine output would be required as biomarkers for AKI. Several biomarkers are reported for AKI, including neutrophil gelatinase-associated lipocalin (NGAL), kidney injury molecule 1 (KIM-1), L-type fatty acid-binding protein (L-FABP), cystatin C, Clusterin, interleukin-18 (IL-18), Proenkephalin A 119–159 (Penkid), the product of insulin-like growth factor binding protein 7 (IGFBP-7), and the tissue inhibitor of metalloproteinase 2 (TIMP-2). However, there is still no gold standard for the early detection of AKI [8,9].

The long-term outcome is also problematic since patients who develop AKI do not necessarily experience a complete recovery of renal function; it may instead lead either to the development of chronic kidney disease (CKD) or to an exacerbation of the rate of progression of preexisting CKD or irreversible ESRD [10]. Kidney cells have the potential to regenerate after AKI [11]. The replacements of detached tubular cells have been analyzed for decades. There are three candidate cell types for regeneration: mesenchymal stem cells (MSCs), kidney stem cells, and remaining tubular cells. It is reported that bone-marrow-derived MSCs transfer to the injured kidneys, differentiate into tubular cells, and become replacements [12]. However, recent gene-tracking systems reveal that replacements by MSCs are less than 1%. Instead, MSCs have an important role in regeneration due to their paracrine effects [13,14]. Adult kidney stem cells have also been explored, and several research groups have identified the role of kidney stem cells in regeneration [15,16,17]. On the other hand, gene-tracking systems reveal that a majority of regenerative cells after AKI were tubular cells that had survived via de-differentiation and gained the stem cell phenotype [18,19]. Further exploration of regenerative mechanisms may lead to a novel therapy for AKI, and microRNAs (miRNAs) are among the important candidates for that. 

miRNAs are non-coding and single-stranded RNAs of 19–23 nucleotides [20]. miRNAs silence their target genes in the post-transcriptional process through 3′-UTR binding. It is also known that miRNAs are involved in a wide range of biological processes, including development, differentiation, cell proliferation, apoptosis, cancer metastasis, inflammation, and fibrosis [20]. miRNAs were discovered in 1993 [21] and confirmed to exist in mammals in 2000 [22]. Currently, more than 2000 types of human miRNAs are registered in miRBase, a database of pre-miRNAs and miRNAs. It is reported that miRNAs work even if they are not necessarily complementary except for 2–8 on the 5′ side [23,24]. It is estimated that more than 60% of human translated genes have at least one conserved miRNA-binding site [25]. It is also suggested that one miRNA has more than one hundred target mRNAs and controls various regulations [26]. Recently accumulated evidence reveals that miRNAs may be the potential biomarkers as well as therapeutic targets for AKI and AKI–CKD transition. In this review article, we summarize the current knowledge about miRNAs as promising biomarkers and potential therapeutic targets for AKI.

## 2. miRNA Production and Dynamics

In the maturation of miRNAs, most primary miRNA transcripts (pri-miRNAs) are transcribed by RNA polymerase II, followed by cleavage by the microprocessor complex Drosha-DiGeorge syndrome critical region 8 (DGCr8) to precursor miRNAs (pre-miRNAs) [27]. After moving to the cytoplasm from the nucleus, the pre-miRNAs are cleaved by Dicer and form a miRNA duplex. The miRNA duplex interacts with Argonaute (AGO) proteins and forms a RNA-induced silencing complex (RISC), finally forming a mature single miRNA while the other strand is degraded. The mature miRNAs target mRNAs in the cytosol, leading to the inhibition of protein translation or mRNA degradation [28]. miRNAs can also be sorted into extracellular vesicles (EVs), such as exosomes, macrovesicles, and apoptotic bodies. These EVs may be secreted, circulating in the blood or urine, transferring into the recipient cells, and acting on them [28]. miRNAs also secrete RNA-binding proteins, such as high-density lipoprotein (HDL) and AGO2. These miRNAs in the blood are called circulating miRNAs [28]. Especially in cancer research, these circulating miRNAs have been reported to be important biomarkers in liquid biopsy [29]. In kidney diseases, miRNAs in serum, plasma, and urine, as well as in kidney tissue, have been explored as potential biomarkers, for example, for nephrotic syndrome, IgA nephropathy, hypertensive nephropathy, diabetic nephropathy, CKD, and AKI [30]. In addition, miRNAs are also explored as therapeutic targets for kidney diseases, including AKI.

## 3. miRNAs as Biomarkers in AKI

### 3.1. Overview of Biomarkers in AKI

Under AKI, the decrease in the glomerular filtration rate leads to an increase in s-Cr after a delay of 24 to 48 h [31]. A renal tubular disorder marker that precedes the s-Cr is thought to be useful for the early diagnosis of AKI. Several tubular injury markers have been reported as AKI biomarkers, including KIM-1, L-FABP, IL-18, NAG, and NGAL, which could indicate kidney damage prior to the increase in s-Cr [32,33]. In addition, cystatin C, clusterin, and penkid have also been reported as the potential biomarkers [34,35]. Among these, NGAL was reported as one of the fastest markers for detecting tubular injury, particularly in the distal tubular segments [34]. In humans, elevated NGAL levels can be observed within 3 h after tubular injury and peak around 6–12 h. It is reported that IL-18 levels rise around 6 h after kidney damage and peak between 12 and 18 h [34]. It is also reported that urinary L-FABP may be elevated 2 h postoperatively in AKI patients, suggesting that the 2 h postoperative urinary L-FABP may predict AKI [34]. It is also reported that G1 cell cycle arrest markers, TIMP-2, and IGFBP7 expressions are increased during the early phase of cellular stress or injury. Indeed, the combination of TIMP2 and IGFBP7 ([TIMP-2] × [IGFBP7]) was reported as an accurate indicator for identifying the early phase of AKI [36]. In spite of these advances in early AKI diagnosis, they are not widely applied in clinical practice. Therefore, novel biomarkers would still be desirable for the early AKI detection and prediction of its severity as well as for the differentiation of AKI etiologies, such as ischemic, drug-induced, contrast-induced, and septic. From this point of view, miRNAs would be potent and novel biomarkers to solve these problems.

### 3.2. miRNAs as AKI Biomarkers

#### 3.2.1. Overview of miRNAs as AKI Biomarkers

A study that analyzed the profile of miRNA in normal human kidneys indicated 669 types of miRNAs, while 364 types of miRNAs were identified in normal mice [37]. Comparing the 20 most abundant types in humans and mice, 12 types of miRNAs (let-7c-5p, miR-10a-5p, miR-10b-5p, miR-143-3p, miR-181a-5p, miR-192-5p, miR-26a-5p, miR-27b-3p, miR-30a-5p, miR-30c-5p, miR-30d-5p, and miR-92a-3p) were held in common. On the other hand, 8 types of miRNAs (let-7a-5p, let-7b-5p, miR-125a-5p, miR-125b-5p, miR-150-5p, miR-191-5p, miR-204-5p, and miR-486-5p) were specific to humans, and 8 types (let-7f-5p, miR-101a-3p, miR-126a-5p, miR-16-5p, miR-21a-5p, miR-22-3p, miR-30e-5p, and miR-378a-3p) were specific to mice [37]. These data suggest that some profile differences exist between the species. In addition, there was another study on the profile of the miRNA Tissue Atlas, where the human miRNA profile, including in kidneys, was analyzed [38]. Currently, there are several profile studies analyzing miRNAs as biomarkers for AKI, where blood or urine samples were applied (Table 1). One of the advantages of miRNA profile analysis is its stability against RNase and low pH as well as multiple freeze–thaw cycles [39]. It was reported that serum levels of a panel of 10 miRNAs (miR-101-3p, miR-127-3p, miR-210-3p, miR-126-3p, miR-26b-5p, miR-29a-3p, miR-146a-5p, miR-27a-3p, miR-93-3p, and miR-10a-5p) may be useful as biomarkers of AKI in ICU, where the panel of miRNAs indicated a high diagnostic value [40]. Among these miRNAs, miR-210-3p, miR-126-3p, miR-29a-3p, and miR-146a-5p were correlated with severity of AKI, diagnosed by AKIN criteria. Urinary miR-16 was 100-fold higher in AKI patients [41]. Similarly, urine miR-16-5p was increased in patients with AKI, suggesting a possible specific biomarker for AKI. On the other hand, plasma miR-16 was down-regulated in patients with AKI [42], indicating a possible difference between blood and urine. In the same study, up-regulation of plasma miR-210 and down-regulation of miR-320 were also reported. Among these, plasma miR-210 was also detected as an independent predictor of 28-day survival [42]. In the other study, urine miR-494 levels were 60-fold higher in patients with AKI [43]. Urine levels of miR-21, miR-200c, and miR-423 were reported to be increased in patients with AKI in ICU, and the combination of these miRNAs showed high AUC at 0.91 [44]. Serum miR-5100 was down-regulated in patients with AKI [45], and urine miR-155 was down-regulated in patients with AKI [46]. In addition, a change in miRNA is often specific to the individual etiologies for AKI.

#### 3.2.2. miRNAs as Biomarkers for Contrast-Induced AKI

As biomarkers for contrast-induced AKI, plasma levels of miR-30a, miR-30c, and miR-30e showed > two-fold increase in patients with contrast-induced nephropathy (CIN) [47]. Another study also indicated increased plasma levels of miR-30a and miR-30b as well as miR-188 in patients with CIN [48], suggesting a specific increase in the plasma miR-30 family in CIN. 

#### 3.2.3. miRNAs as Biomarkers for Sepsis-Induced AKI

As biomarkers for sepsis-induced AKI, serum levels of miR-29a and miR-10a-5p were increased in patients with sepsis-induced AKI, and these miRNAs were also detected as the predicting marker for 28-day survival [49]. Urine miR-26b was also reported to be increased in patients with sepsis-induced AKI and was associated with mortality [50]. Increased plasma miR-494 was also reported to predict 28-day survival in patients with sepsis-induced AKI [51]. Serum miR-21 was also up-regulated in patients with sepsis-induced AKI [52]. It was also reported that serum and urine miR-22-3p was down-regulated in sepsis-induced AKI patients, and may serve as a biomarker to predict 28-day survival [53]. Regarding the early detection of AKI, increases in serum and urine levels of miR-452 in patients with sepsis-induced AKI were reported, where the sensitivity of urine miR-452 was higher than urine [TIMP-2] × [IGFBP7] [54].

#### 3.2.4. miRNAs as Biomarkers for AKI Caused by Cardiac Surgery

As biomarkers for AKI caused by cardiac surgery, urine miR-30c-5p as well as miR-192-5p were increased in patients with AKI after cardiac surgery as early as 2 h post operation, suggesting a possible biomarker for predicting AKI after cardiac surgery [55]. Similarly, plasma miR-192 was also increased in patients with AKI at the time of ICU admission, remaining stable for 2 h and decreasing after 24 h, suggesting a time-dependent change in miR-192 [56]. Levels of serum, plasma and urine miR-21 were increased in patients with AKI after cardiac surgery [57,58]. In addition, urine and plasma miR-21 levels also predicted the need for post-operative renal replacement therapy, 30-day in-hospital mortality, and prolonged stay in hospital or ICU [58]. Serum miR-494 was up-regulated in child patients with AKI after cardiopulmonary bypass for congenital heart disease. In the report, the combination of NGAL, Kim-1, and miR-494 showed the high area under the curve (AUC) to predict the death of children with postoperative AKI [59]. On the other hand, decreased miR-21 levels in patients with AKI after cardiac surgery were also reported [60], where reduced post-operative serum and urine miR-21 levels could predict AKI development. In addition, it is also reported that baseline miR-21 before cardiac surgery could predict AKI development [61]. These results may reflect the complex regulation of miR-21 under AKI. 

#### 3.2.5. Summary of miRNAs as Biomarkers for AKI

In summary, there are several promising miRNAs that could serve as possible biomarkers for early diagnosis, prediction of mortality, and the specific pathology in AKI. Nevertheless, there are several limitations and problems relating to clinical use. For example, several types of samples, such as serum, plasma, urine, and exosomal miRNAs, may be used for the analysis. Indeed, it is reported that serum and plasma may yield result differences. In addition, the collecting methods of miRNAs are different between reports, which may be one cause of the differences between the reports. Since miRNA volume is very limited, the methods of analysis and collection of EV may affect the results. Therefore, clinical procedures still need exploration.

**Table 1:** miRNAs as potential biomarkers of AKI.

miRNA	Subtype	Species	Etiology	Expression	Sample	Ref.
miR-30	miR-30a, c, e	Human/rats	Contrast	Up	Plasma	[47]
	miR-30a, e	Human/rats	Contrast	Up	Plasma	[48]
	miR-30c-5p	Human	Cardiac surgery	Up	Urine	[55]
miR-188	miR-188	Human	Contrast	Up	Plasma	[48]
miR-22	miR-22-3p	Human	Sepsis	Down	Serum/Urine	[53]
miR-29	miR-29a	Human	Sepsis	Up	Serum	[49]
miR-26	miR-26b	Human	Sepsis	Up	Urine	[50]
miR-10	miR-10a-5p	Human	Sepsis	Up	Serum	[49]
miR-452	miR-452	Human	Sepsis	Up	Serum/Urine	[54]
miR-210	miR-210	Human	-	Up	Plasma	[42]
	miR-210	Human	Sepsis	Up	Plasma	[51]
miR-494	miR-494	Human/mice	-	Up	Urine	[43]
	miR-494	Human	Cardiac surgery	Up	Serum	[59]
	miR-494	Human	Sepsis	Up	Plasma	[51]
miR-21	miR-21	Human	Cardiac surgery	Down	Serum/Urine	[60]
	miR-21	Human	Cardiac surgery	Up	Plasma/Urine	[58]
	miR-21	Human	Cardiac surgery	Up	Serum/Urine	[57]
	miR-21-3p	Human	Sepsis	UP	Serum	[52]
	miR-21	Human	-	Up	Urine	[44]
	miR-21	Human	-	Up	Urine	[46]
miR-192	miR-192-5p	Human	Cardiac surgery	Up	Urine	[55]
	miR-192	Human/rats	Cardiac surgery	Up	Plasma	[56]
miR-16	miR-16	Human/mice	-	Up	Urine	[41]
	miR-16	Human	-	Down	Plasma	[42]
	miR-16-5p	Human	-	Up	Urine	[62]
miR-155	miR-155	Human	-	Down	Urine	[46]
miR-200	miR-200c	Human	-	Up	Urine	[44]
miR-423	miR-423	Human	-	Up	Urine	[44]
miR-5100	miR-5100	Human	-	Down	Serum	[45]
miR-320	miR-320	Human	-	Down	Plasma	[42]

miRNA: microRNA.

## 4. miRNAs as Therapeutic Targets for AKI

### 4.1. Overview of Treatment in AKI

Treating AKI is the ultimate challenge. According to the Kidney Disease: Improving Global Outcomes (KDIGO) Clinical Practice Guideline for Acute Kidney Injury [63], there is no strong recommendation for its administration; thus, there is a demand for the development of new AKI therapeutic agents. As regeneration of kidneys can occur, there are several challenges involved. For example, stem cell therapy, including mesenchymal stem cells, kidney stem cells, and induced pluripotent stem (iPS)-cell-derived nephron progenitor cells, has been explored over several decades [14,64,65]. There are reports on direct replacement of transplanted stem cells [15,66], but currently, it appears likely that the dominant effect of transplanted stem cells is derived from the secreted factors from stem cells. For example, MSCs secrete a variety of factors, including soluble factors, cytokines, chemokines, and growth factors [14]. In addition, MSCs secrete extracellular vesicles containing soluble proteins, mRNAs, and miRNAs [13]. These factors transfer to the recipient cells, which mediate renoprotection (anti-apoptosis, anti-nectrosis, anti-inflammation, anti-oxidative stress, and anti-fibrosis) and regeneration (cell proliferation, cell migration, tubular de-differentiation, and angiogenesis) [67]. It is likely that extracellular vesicles are the predominant paracrine effects in AKI [67]. Which factors provide the dominant therapeutic effects? It was reported that EVs derived from MSCs with knockdown of Drosha, essential for miRNA production, failed to ameliorate I/R-induced AKI, while MSC-derived EVs without knockdown ameliorated AKI [68]. These data suggest that miRNAs in EVs might be the most important factors for renal protection and regeneration in AKI. Renoprotective miRNAs from MSCs were previously reported [67]. In the report, miR-21 and miR-30 mediated anti-apoptosis, miR-210 mediated angionegesis, miR-145 mediated autophagy, miR-15 and miR-16 ameliorated kidney fibrosis, and miR-15, miR-16, miR-21, and let-7 ameliorated inflammation through the regulation of macrophage. In addition to MSCs, secreted factors from other stem cells, such as kidney stem cells and iPS-derived nephron progenitor cells, have also been reported to be renoprotective [14,64,65]. These trophic effects might be at least partly delivered via miRNAs. miRNAs from other cells, such as circulating inflammatory cells and tubular cells, might be involved in kidney damage and/or regeneration during AKI. Researchers still need to explore miRNA dynamics. Nevertheless, miRNAs might be novel therapeutic targets for AKI.

In addition, a hypoxia-inducible factor (HIF)-prolyl hydroxylase (HIF-PHD) inhibitor has been developed as a therapy for renal anemia [69]. The pharmacological activation of HIF regulates a variety of genes, including Epo, leading to hematopoiesis. Other than hematopoiesis, the HIF-PHD inhibitor has also been shown to ameliorate AKI in rodent experimental models [70], including I/R-induced and drug-induced models with cisplatin, gentamicin, and lipopolysaccharide (LPS). These protective mechanisms include anti-apoptosis via miR-21, anti-inflammation by macrophage reduction, reduced VCAM1, up-regulation of angiogenesis via VEGF up-regulation, and anti-oxidative stress via up-regulation of Heme Oxygenase 1 (HO-1) [70]. As shown with increases in miR-21, these effects may be mediated at least partly via regulation of miRNAs. Indeed, it is reported that kidney ischemia activates HIF-1*α*, which in turn up-regulates miR-21, leading to anti-apoptosis through the suppression of pro-apoptotic factor programmed cell death protein 4 (Pdcd4) and phosphatase and tensin homolog deleted from chromosome 10 (PTEN) [71]. HIF1 also increases miR-668 expression, which targets mitochondrial fission process protein 1 (MTP18), leading to the protection of kidney tubular cells via mitochondrial dynamics under ischemic AKI in humans and mice [72]. Activation of HIF also increases miR-489, leading to anti-apoptosis in kidney tubular cells during ischemic AKI though targeting repair sensor poly(ADP-ribose) polymerase 1 (PARP1) [73]. Taken together, these data suggest that the regulation of miRNA might be a novel and specific therapy against AKI.

### 4.2. Therapeutic Targeting of miRNAs for AKI

#### 4.2.1. Overview of Therapeutic miRNA Target for AKI

Some of the most important evidence regarding miRNAs in AKI was reported in 2010, where knockouts of tubular miRNAs were analyzed in the rodent model [74]. The loxp-cre system was used to produce mice lacking Dicer, a key enzyme for miRNA production, predominantly in kidney proximal tubular cells by crossing with phosphoenolpyruvate carboxykinase (PEPCK)-cre mice. The mice showed global down-regulation of miRNAs in the kidney cortex and had normal kidney function and histology under normal conditions. Importantly, the mice were resistant to renal ischemia-reperfusion (I/R) injury, demonstrating the involvement of miRNAs under AKI. Since then, there has been accumulating evidence supporting miRNAs as potential therapeutic targets in AKI. Several miRNAs have been reported to have protective and/or pathogenic roles in AKI, regulating tubular apoptosis, tubulointerstitial fibrosis, and inflammation in a variety of etiologies of AKI, including ischemia, drug, and sepsis. Some miRNAs have common gene targets. For example, miR-30 and miR-26a target Snai1, regulating epithelial–mesenchymal transition (EMT), while miR-21, miR-17, miR-188, and miR-378 target PTEN, which is implicated in cell apoptosis, proliferation, inflammation, and fibrosis [27]. The potential therapeutic targets of miRNAs for AKI are summarized in Table 2.

#### 4.2.2. Protective miRNAs for AKI

There are several protective mechanisms of miRNAs under AKI, including regulation of cell apoptosis, inflammation, autophagy, and energy metabolism. For example, miR-30, miR-181, miR-22, and miR-590 are reported to mediate anti-apoptosis and anti-inflammation effects in I/R-AKI rodent models. It was reported that MSC-derived extracellular vesicles ameliorated rat I/R-induced AKI by inhibiting cell apoptosis through miR-30b, miR-30c, and miR-30d, which targeted dynamin-related protein 1 (DRP1), thereby inhibiting mitochondrial fission [75]. In addition, injection of miR-30c-5p agomir, a chemically modified double-stranded small RNA that mimics the miR-30c-5p, ameliorated rat I/R-induced AKI through transformation of M1 to M2 macrophages, mediating an anti-inflammatory effect via changes in inflammatory cytokines [76]. miR-181d-5p overexpression in the mouse model of I/R-induced AKI ameliorated kidney injury by reducing inflammatory mediators and apoptosis through targeting Krueppel-like factor 6 (KLF6) [77]. A renoprotective effect of miR-181 was also shown in a cisplatin-induced mouse AKI model by targeting PTEN [78], as well as in a LPS-induced AKI model through an anti-apoptosis effect by targeting GJB2 [79]. In addition, miR-181a-5p is reported to target NIMA-related kinase 7 (NEK7), thereby inhibiting pyroptosis in sepsis-induced AKI mice [80]. It was reported that in a sepsis-induced AKI mouse model, lncRNA TCONS_00016233, targeting miR-22-3p, was up-regulated in plasma. In the report, TCONS_00016233 overexpression worsened sepsis-induced mouse AKI by down-regulating miR-22-3p, thus increasing the miR-22-3p target, apoptosis-inducing factor mitochondrion-associated 1 (AIFM1), leading to apoptosis [81]. These data suggest an anti-apoptosis effect of miR-22. In addition, miR-22 attenuated sepsis-induced rat AKI, targeting High Mobility Group Box 1 (HMGB1) and inhibiting the HMGB1/TLR4/NF-kB pathway [82]. Adenovirus expressing miR-590-3p via a tail-vein injection in LPS-induced septic AKI mice ameliorated cell apoptosis and inflammation by targeting tumor necrosis factor receptor-associated factor 6 (TRAF6) [83]. miR-590-3p was also shown to possibly increase autophagy and protect kidney injury by targeting TRAF6, which was evaluated in an in vitro I/R model using renal tubular epithelial cell line (HK-2 cells) [84]. 

There are several reports indicating an anti-apoptosis effect from miRNAs. miR-124 was shown to ameliorate I/R-induced mouse AKI, where miR-124 mimics reduced endoplasmic reticulum stress (ERS)-mediated apoptosis [85]. In addition to the anti-apoptosis mechanism, miR-124 also inhibited necroptosis by targeting PARP1 in an I/R-induced mouse AKI model [86]. miR-489 also targeted PARP1, mediating an anti-apoptosis effect, and miR-489 mimics protected against I/R mouse AKI [73]. miR-17-5p mimics suppressed death receptor 6 (DR6), mediating anti-apoptosis in an I/R-induced mouse AKI model [87]. Likewise, miR-424 mimics inhibited its target gene DR6, mediating anti-apoptosis in an I/R-induced mouse AKI model [88]. A miR-5100 mimic injection into I/R-mice ameliorated kidney injury by inhibiting several apoptotic pathways [45]. miR-486-5p was shown to target PTEN, thereby mediating an anti-apoptosis effect in a mouse I/R-AKI model [89]. A miR-191-5p mimic injection could inhibit cell apoptosis by targeting Oxidative stress responsive 1 (OXSR1) [90]. miR-290-5p activated by propofol ameliorated a sepsis-induced mouse AKI model by targeting C-C motif chemokine ligand 2 (CCL2), thereby mediating an anti-apoptosis effect [91]. 

In addition to the anti-apoptosis effect, an anti-inflammation effect by miRNAs has also been explored. miR-204/miR-211 mimics, targeting H6 Family Homeobox 1 (Hmx1), reduced kidney injury via immune suppression in candidemia-induced AKI mice [92]. A miR-195-5p mimic injection ameliorated rat I/R-AKI by targeting vascular endothelial growth factor A (VEGFA) via anti-inflammatory and anti-oxidative stress [93]. miR-140-5p up-regulation by apigenin ameliorated I/R-induced AKI mice through targeting Chemokine (C-X-C Motif) Ligand 12 (CXCL2), thereby reducing inflammation [94]. In addition, miR-140-5p was shown to activate nuclear factor erythroid 2-related factor (Nrf2) pathway, thereby mediating anti-oxidative stress in cisplatin-induced AKI mice [95]. miR-27a targeting Toll-like receptor 4 (TLR4) inhibited inflammation in I/R-induced AKI [96]. In addition, overexpression of LINC00520, targeting miR-27b-3p, activated Oncostatin M Receptor (OSMR), leading to the PI3K/AKT pathway to aggravate kidney injury in I/R-induced AKI. In contrast, up-regulation of miR-27b-3p could accelerate recovery from AKI [97]. miR-146a-5p derived from human urine-derived stem cells protected against rat I/R-induced AKI by targeting interleukin 1 receptor associated kinase 1 (IRAK1), thereby inhibiting NF-κB signaling and infiltration of inflammatory cells [98]. Mice lacking miR-146 showed more extensive tubular injury, inflammatory infiltrates, and fibrosis than wild-type mice after I/R-induced AKI [99]. Furthermore, the renoprotective effect of miRNAs involves vascular, mitochondrial, and podocyte protection. miR-210 was shown to activate VEGF signaling to regulate angiogenesis in I/R-induced AKI mice [100]. Overexpression of miR-126 in the hematopoietic compartment promoted vascular integrity and supported the recovery of kidney injury after I/R-induced AKI mice [101]. miR-668 was shown to target mitochondrial protein 18 kDa (MTP18) to preserve mitochondrial dynamics and tubular cell survival in I/R-AKI mice [72]. A miR-187 agomir injection in I/R protected against I/R-induced AKI and mediated podocyte protection, evaluated by nephrin expression by targeting acetylcholinesterase (AChE) [102]. 

There are several reports indicating MSC-related miRNAs as providing renoprotection via anti-apoptosis. MSC-derived exosome containing miR-199a-3p targeted semaphorin3A (SEMA3A), leading to the AKT and Extracellular signal-regulated kinase (ERK) pathway activation and ameliorated apoptosis [103]. Bone-marrow-derived MSCs ameliorated I/R-induced mouse AKI via miR-223, targeting NLR family pyrin domain containing 3 (NLRP3), thereby inhibiting apoptosis [104]. Bone-marrow-derived MSCs ameliorated cisplatin-induced AKI by inhibiting fibrosis through the regulation of miR-146a-5p and its target transcription Factor Dp-2 (Tfdp2) [105].

#### 4.2.3. Pathogenic miRNAs for AKI

In addition to these renoprotective miRNAs, there are several pathogenic miRNAs under AKI, aggravating kidney injury by causing apoptosis, inflammation, mitochondrial damage, and fibrosis. It is reported that the administration of an antisense oligonucleotide inhibiting miR-182-5p ameliorated an I/R-induced AKI rat model [106]. In another report, an miR-182 inhibitor ameliorated I/R-induced rat AKI and apoptosis by regulating the transcription factor 7-like-2 (TCF7L2)/ Wnt/β-catenin pathway [107]. miR-182 was also shown to target Forkhead box O3 (FoxO3), leading to cell apoptosis in an I/R-induced AKI rat model [108]. Similarly, there are several miRNAs reported to promote cell apoptosis under AKI, including miR-122, miR-301, miR-375, miR-188, miR-687, miR-24, and miR-218. miR-122 was suggested to target FoxO3, thereby causing cell apoptosis in cisplatin-induced AKI mice [109]. miR-301a-5p inhibition ameliorated vancomycin-induced AKI by reducing apoptosis [110]. miR-375 was reported to be induced in a cisplatin-induced mouse AKI model to repress hepatocyte nuclear factor 1 homeobox B (HNF-1β), leading to the promotion of tubular cell apoptosis [111]. miR-188 was shown to aggravate contrast-induced AKI by targeting Serine and Arginine Rich Splicing Factor 7 (SRSF7), leading to cell apoptosis [112]. miR-687 was shown to be interfered with by lncRNA TCONS_00016406, leading to an anti-apoptosis effect [113]. miR-24 silencing protected against I/R-induced mouse AKI by inhibiting cell apoptosis by targeting H2A histone family, member X, (H2A.X), and HO-1 [114]. It was also suggested that honokiol inhibited miR-218-5p, leading to an increase in its target HO-1, thereby mediating an anti-apoptosis effect in sepsis-induced mouse AKI [115]. 

Several miRNAs, namely, miR-494, miR106, miR-155, and miR-152, were shown to promote inflammation as well as apoptosis. It is reported that miR-494-3p down-regulation by lncRNA TUG1 reduced I/R-induced mouse AKI and cell apoptosis by regulating E-cadherin [116]. In addition, overexpression of miR-494 was shown to reduce activating transcription factor 3 (ATF3), leading to an increase in inflammatory mediators, such as IL-6 and monocyte chemotactic protein-1, exacerbating inflammation in I/R-induced mouse AKI [43]. An injection of miR-494 antagomir, a chemically modified miR-494 antagonist, ameliorated LPS-induced mouse AKI via anti-apoptosis and anti-inflammation mechanisms by regulating the NF-κB signaling pathway [117]. It was reported that serum miR-106a was increased in sepsis-induced AKI mice, and miR-106a was suggested to target thrombospondin 2 (THBS2), leading to inflammation and apoptosis [118]. miR-155 inhibitor ameliorated LPS-induced mouse AKI through the reduction in inflammatory cells by regulating the target Suppressor Of Cytokine Signaling 1 (SOCS1) and Signal Transducer And Activator Of Transcription (STAT)1 mRNAs [119]. It was also reported that macrophage-derived exosomal miR-155 promoted tubular injury by targeting SOCS1 [120]. In addition, miR-155^−/−^ mice were made resistant to cisplatin-induced AKI by reducing tubular cell apoptosis [120]. miR-155 was shown to be up-regulated in rat I/R-induced AKI, and suggested to promote kidney injury and apoptosis by targeting Transcription factor 4 (TCF4)/Wnt/β-catenin signaling pathway [121]. miR-152-3p was suggested to promote cell apoptosis by silencing Sirtuin 7 (SIRT7) in I/R-induced rat AKI [122]. miR-152-3p was shown to promote sepsis-induced rat AKI by targeting ERBB receptor feedback inhibitor 1 (ERRFI1), leading to an increase in STAT3 expression, resulting in promoting cell apoptosis and inflammation [123]. An miR-709 antagomir injection attenuated cisplatin-induced mouse AKI via a reduction in mitochondrial dysfunction by regulating miR-709 target gene mitochondrial transcriptional factor A (TFAM) [124].

#### 4.2.4. miRNAs with Both Protective and Pathogenic Effects for AKI

miR-21 is one of the most analyzed miRNAs, described as having double-edged-sword effects in kidneys [67] and both protective and pathogenic effects in kidney diseases. As a protective effect, miR-21 ameliorates I/R-induced AKI by inhibiting tubular cell apoptosis in I/R- and LPS-induced AKI mice [71,125], targeting PTEN/Akt/mammalian target of rapamycin (mTOR) signaling and Cyclin-dependent kinase 6 (CDK6). miR-21 is also shown to inhibit maturation of dendritic cells through the PDCD4/ NFκ-B pathway [71] and CCR7 [126], thereby mediating anti-inflammatory effects in I/R-induced AKI mice. In addition, miR-21 is also shown to target mitogen-activated protein kinase kinase 3 (MKK3), inhibiting the downstream factors IL-6 and TNF-α levels, mediating anti-inflammatory effects [127]. On the other hand, as a pathogenic effect, miR-21 inhibits autophagy in I/R-AKI rats by targeting Rab11a [128]. In addition, long-term elevation of miR-21 might promote kidney fibrosis, including via PPARα [129]. Furthermore, miR-21-3p was shown to regulate energy metabolism via AKT/Cyclin-dependent kinase 2 (CDK2)-FOXO1 in a sepsis-induced rat AKI model, while it was unclear whether the regulation was protective or harmful for a long-term prognosis [130]. Taken together, these data suggest that miR-21 may target several signaling pathways, involving cell apoptosis, inflammation, and autophagy, as well as energy metabolism under AKI. 

Similarly to miR-21, several miRNAs were shown to mediate both protective and pathogenic effects under AKI. A miR-34b-3p agomir injection ameliorated sepsis-induced mouse AKI via an anti-inflammatory mechanism by targeting ubiquitin-like protein 4A (UBL4A) [131]. miR-34a induced via p53 was suggested to play a cytoprotective role in cell survival in cisplatin-induced mouse AKI [132]. In contrast to these renoprotective effects of miR-34, miR-34 was also shown to promote kidney injury under AKI. lncRNA HOX transcript antisense RNA (HOTAIR) overexpression ameliorated sepsis-induced rat AKI via an anti-apoptosis mechanism by targeting miR-34a and regulating its target B-cell lymphoma-2 (Bcl-2) [133]. It is also reported that increased miR-34a promoted acetylation of FOXO3 by repressing Sirtuin 1 (SIRT1), leading to p53 activation and cell apoptosis in the cisplatin-induced mouse AKI model [109]. miR-34a was also suggested to suppress autophagy in kidney tubular cells by targeting autophagy-related 4B cysteine peptidase (ATG4B) in I/R-induced mouse AKI [134]. miR-34a mimics prevented Nicotinamide phosphoribosyltransferase (NAMPT) expression, which suggest that they affected oxidized NAD (NAD^+^) metabolism, leading to kidney dysfunction in I/R-induced AKI mice [135]. Human umbilical-cord-derived MSCs containing miR-125b-5p suppressed p53, leading to an anti-apoptosis effect in I/R-induced AKI mice [136]. In contrast, miR-125 was also shown to disrupt mitochondrial dynamics by targeting modulate mitofusin1 (MFN1) in cisplatin-induced AKI mice, where anti-miR-125b treatment reduced cisplatin-induced mitochondrial fragmentation and kidney injury [137]. miR-150-5p agomir was shown to ameliorate sepsis-induced mouse AKI via an anti-apoptosis mechanism by targeting mitogen-activated protein kinase kinase 3 (MEKK3)/JNK pathway [138]. On the other hand, it was also reported that the deletion of miR-150 in mice protected against myocardial-infarction-induced AKI through an anti-apoptosis and anti-fibrosis mechanism by targeting insulin-like growth factor-1 receptor (IGF-1R) [139]. In addition, pretreatment with exosomes enriched in miR-150 worsened kidney fibrosis in I/R-induced AKI mice [140]. Similarly, kidney fibrosis was reduced by miR-150-5p-deficient tubular-cell-derived exosome in I/R-induced AKI mice by regulating the miR-150 target gene, SOCS1 [141]. miR-214 is also shown to mediate both protective and pathogenic effects under AKI. Regarding renoprotective effects, a miR-214 injection in I/R-induced mice ameliorated kidney injury by inhibiting apoptosis through targeting Dickkopf WNT signaling pathway inhibitor 3 (Dkk3) [142]. Adenovirus-mediated miR-214 treatment inhibited excess autophagy through regulation of the PTEN/AKT/mTOR pathway, thereby limiting kidney injury in sepsis-induced AKI mice [143]. On the other hand, regarding pathogenic effects, it is reported that kidney proximal-tubular-cell-specific miR-214 knockout mice showed less kidney damage and less apoptosis after I/R-induced AKI by targeting mitofusin-2 (Mfn2) [144]. miR-214-5p antagomir ameliorated LPS-induced kidney inflammation and oxidative stress, while miR-214-5p agomir aggravated kidney injury, presumably by targeting glucagon-like peptide-1 receptor (GLP-1R) [145]. miR-214-3p antagomir protected against cisplatin-induced AKI in mice by inhibiting tubular cell ferroptosis by targeting Glutathione Peroxidase 4 (GPX4) [146]. Microvesicles from human Wharton’s Jelly MSCs ameliorated I/R-induced rat AKI by suppressing C-X3-C Motif Chemokine 1 (CXCL1), and ameliorated inflammation and fibrosis partly via miR-16, miR-15a, and miR-15b [147]. On the other hand, urine miR-16 transactivated by CCAAT enhancer binding protein beta (C/EBP-β) worsened I/R-induced AKI, causing apoptosis [41]. Bone-marrow-derived MSCs reduced miR-107, increasing the expression of the miR-107 target, Ribosomal Protein S19 (RPS19), and protected from cisplatin-induced apoptosis [148]. In contrast, miR-107 was shown to induce TNF-α by targeting dual specificity phosphatase 7 (DUSP7), causing tubular injury [149]. Collectively, miRNAs have several gene targets, thus regulating a variety of mechanisms that may mediate protective and/or pathogenic effects under AKI, and it may depend on the etiologies of AKI and evaluating methods.

**Table 2:** Potential therapeutic targets of miRNAs for AKI.

miRNA	Effect	Target	Model	Species	Function	Ref.
miR-30	Protective	DRP1	I/R	Rats	Anti-apoptosis	[75]
	Protective	M1-M2 macrophage transition	I/R	Rats	Anti-inflammation	[76]
miR-181	Protective	KLF6	I/R	Mice	Anti-apoptosis Anti-inflammation	[77]
	Protective	PTEN	Cisplatin	Mice	-	[78]
	Protective	GJB2	LPS	Mice	Anti-apoptosis	[79]
miR-22	Protective	AIFM1	Sepsis	Mice	Anti-apoptosis	[81]
	Protective	HMGB1	Sepsis	Rats	Anti-inflammation	[82]
miR-590	Protective	TRAF6	LPS	Mice	Anti-apoptosis Anti-inflammation	[83]
miR-124	Protective	PARP1	I/R	Mice	Anti-necroptosis	[86]
	Protective	-	I/R	Mice	Anti-apoptosis	[85]
miR-489	Protective	PARP1	I/R	Mice	Anti-apoptosis	[73]
miR-17	Protective	DR6	I/R	Mice	Anti-apoptosis	[87]
miR-424	Protective	DR6	I/R	Mice	Anti-apoptosis	[88]
miR-5100	Protective	Apoptotic pathway	I/R	Mice	Anti-apoptosis	[45]
miR-486	Protective	PTEN	I/R	Mice	Anti-apoptosis	[89]
miR-191	Protective	OXSR1	Sepsis	Rats	Anti-apoptosis	[90]
miR-290	Protective	CCL2	Sepsis	Mice	Anti-apoptosis	[91]
miR-204	Protective	Hmx1	Candidemia	Mice	Anti-inflammation	[92]
miR-211	Protective	Hmx1	Candidemia	Mice	Anti-inflammation	[92]
miR-195	Protective	VEGFA	I/R	Rats	Anti-inflammation Anti-oxidative stress	[93]
miR-140	Protective	Nrf2	Cisplatin	Mice	Anti-oxidative stress	[95]
	Protective	CXCL12	I/R	Mice	Anti-inflammation	[94]
miR-27	Protective	OSMR	I/R	Rats	PI3K/AKT signal	[97]
	Protective	TLR4	I/R	Rats	Anti-inflammation	[96]
miR-146	Protective	IRAK1	I/R	Rats	Anti-inflammation	[98]
	Protective	-	I/R	Mice	Anti-inflammation	[99]
	Protective	Tfdp2	Cisplatin	Mice	Anti-fibrosis	[105]
miR-210	Protective	VEGF pathway	I/R	Mice	Angiogenesis	[100]
miR-126	Protective	-	I/R	Mice	Vascular protection	[101]
miR-668	Protective	MTP18	I/R	Mice	Mitochondrial dynamics	[72]
miR-187	Protective	AChE	I/R	-	Podocyte protection	[102]
miR-199	Protective	Sema3A/AKT/ERK	I/R	Mice	Anti-apoptosis	[103]
miR-223	Protective	NLRP3	I/R	Mice	Anti-apoptosis	[104]
miR-182	Pathogenic	-	I/R	Rats	-	[106]
	Pathogenic	TCF7L2/Wnt/β-catenin	I/R	Rats	Apoptosis	[107]
	Pathogenic	FoxO3	I/R	Rats	Apoptosis	[108]
miR-122	Pathogenic	FoxO3	Cisplatin	Mice	Apoptosis	[109]
miR-301	Pathogenic	-	Vancomycin	Mice	Apoptosis	[110]
mR-375	Pathogenic	HNF1b	Cisplatin	Mice	Apoptosis	[111]
miR-188	Pathogenic	SRSF7	Contrast	Rats	Apoptosis	[112]
miR-687	Pathogenic	-	LPS	Mice	Apoptosis	[113]
miR-24	Pathogenic	H2A.X/HO-1	I/R	Mice	Apoptosis	[114]
miR-218	Pathogenic	HO-1	Sepsis	Mice	Apoptosis	[115]
miR-494	Pathogenic	ATF3	I/R	Mice	Inflammation	[43]
	Pathogenic	E-cadherin	I/R	Mice	Apoptosis	[116]
	Pathogenic	NF-κB signaling	LPS	Mice	Inflammation Apoptosis	[117]
miR-106	Pathogenic	THBS2	Sepsis	Mice	Inflammation Apoptosis	[118]
miR-155	Pathogenic	SOCS1/STAT1	LPS	Mice	Inflammation	[119]
	Pathogenic	TCF4/Wnt/β-catenin	I/R	Rats	Apoptosis	[121]
	Pathogenic	SOCS1	I/R	Mice	Tubular injury	[120]
	Pathogenic	-	Cisplatin	Mice	Apoptosis	[150]
miR-152	Pathogenic	ERRFI1	Sepsis	Rats	Inflammation Apoptosis	[123]
	Pathogenic	SIRT7	I/R	Rats	Apoptosis	[122]
miR-709	Pathogenic	TFAM	Cisplatin	Mice	Mitochondrial damage	[124]
miR-21	Protective	PTEN/Akt/mTOR Pdcd4/NFκ-B	I/R	Mice	Anti-apoptosis Anti-inflammation	[71]
	Protective	MKK3	I/R	Mice	Anti-inflammation	[127]
	-	AKT/CDK2-FOXO1	Sepsis	Rats	Metabolism alteration	[130]
	Protective	CDK6	LPS	Mice	Anti-apoptosis	[125]
	Protective	CCR7	I/R	Mice	Anti-inflammation	[126]
	Pathogenic	Rab11a	I/R	Rats	Anti-autophagy	[128]
miR-34	Pathogenic	NAMPT	I/R	Mice	NAD depletion	[135]
	Pathogenic	Atg4B	I/R	Mice	Reduced autophagy	[134]
	Pathogenic	Bcl2	Sepsis	Rats	Apoptosis	[133]
	Pathogenic	SIRT1	Cisplatin	Mice	Apoptosis	[109]
	Protective	UBL4A	Sepsis	Mice	Anti-inflammation	[131]
	Protective	-	Cisplatin	Mice	Cytoprotective	[132]
miR-125	Pathogenic	MFN1	Cisplatin	Mice	Mitochondrial damage	[137]
	Protective	P53	I/R	Mice	Anti-apoptosis	[136]
miR-150	Pathogenic	IGF-1R	Ischemic	Mice	Apoptosis Fibrosis	[139]
	Pathogenic	-	I/R	Mice	Fibrosis	[140]
	Pathogenic	SOCS1	I/R	Mice	Fibrosis	[141]
	Protective	MEKK3/JNK	LPS	Mice	Anti-apoptosis	[138]
miR-214	Pathogenic	Mfn2	I/R	Mice	Apoptosis	[144]
	Pathogenic	GLP-1R	LPS	Mice	Inflammation	[145]
	Pathogenic	GPX4	Cisplatin	Mice	Ferroptosis	[146]
	Protective	PTEN/AKT/mTOR	Sepsis	Mice	Autophagy regulation	[143]
	Protective	Dkk3	I/R	Mice	Anti-apoptosis	[142]
miR-15	Protective	CX3CL1	I/R	Rats	Anti-inflammation Anti-fibrosis	[147]
miR-16	Protective	CX3CL1	I/R	Rats	Anti-inflammation Anti-fibrosis	[147]
	Pathogenic	-	I/R	Mice	Anti-apoptosis	[41]
miR-107	Protective	RPS19	I/R	Rats	Anti-apoptosis	[148]
	Pathogenic	DUSP7	Sepsis	Mice	Tubular injury	[149]

miRNA: microRNA; I/R: ischemia-reperfusion; LPS: lipopolysaccharide.

### 4.3. Therapy Targeting miRNAs

Nucleic acid drugs can treat adults at the level of RNA. They are usually composed of oligonucleic acids linked by ten to several tens of bases, and act directly on the body without gene expression [151,152]. Nucleic acid drugs include antisense, siRNA, miRNA, decoy, aptamer, and CpG oligodeoxynucleotides [153]. Among these, two approaches can be used for targeting miRNAs: antisense for inhibition-specific miRNA and miRNA-mimic injection. Clinical trials targeting miRNAs as nucleic acid medicine are currently still limited. Anti-miR was first conducted with miR-122 with locked nucleic acid in the form of Miravirsen for treating type C hepatitis [154]. Subsequently, antisense miR-155 (MRG-106) for T-cell lymphoma and mycosis fungoides was tested in a clinical trial [155]. A miR-10b antisense was used in preclinical trials with dextran-coated iron oxide nanoparticles for multiple cancers [156,157,158,159]. miRNA mimic treatment was conducted with miR-34 in lipid nanoparticles in the form of MRX34 for targeting multiple cancers, including hepatic cancers [160,161,162]. Subsequently, a clinical trial (Phase 1) with miR-16 mimics (TargomiR) was conducted for mesothelioma and lung cancer using the bacterial minicell EnGeneIC delivery system [163]. 

Currently, treatment targeting miRNAs in kidney diseases in the clinical stage is limited. A phase 2 placebo-controlled randomized controlled trial for Alport syndrome (NCT02855268) using oligonucleotides of miR-21 is currently ongoing [164], where the efficacy for the progression of kidney dysfunction, as well as the safety, pharmacodynamics, and pharmacokinetics, are being evaluated. A phase 1 clinical trial in polycystic kidney disease (PKD) patients using anti-miR-17 oligonucleotide RGLS4326 is also being conducted (NCT04536688) [165], where the primary objective is to assess the dose–response relationship between RGLS4326 and biomarkers of PKD. Referring to the miRNA treatment for AKI, miR-5100 was recently detected as a potential AKI biomarker as well as a therapy target [45]. miR-5100 was down-regulated in a rodent AKI model, and a miR-5100 mimic ameliorated I/R-induced AKI. In addition, in human serum samples, miR-5100 was down-regulated; thus, it is possibly both a biomarker and a therapy target. 

In spite of these potentials of miRNAs as the therapeutic targets under AKI, there are several limitations and problems relating to clinical use. One of the most important challenges for miRNA-targeting therapy is the use of a drug delivery system (DDS) to transfer these anti-miR or miR-mimics more efficiently. Antisense drugs composed of single-stranded oligonucleic acids must be translocated into cells because they function by complementary binding to intracellular RNA. However, the antisense is large in size and has a negative charge due to the phosphodiester bond, making it difficult for it to pass through biological membranes. Therefore, many antisense miRNAs are attached with a phosphorothioate modification (Sylation). In addition, chemical modifications are also introduced into the sugar moiety of nucleic acids, thereby exerting efficacy without using carriers, such as liposomes. In contrast, miRNA mimics are normally composed of a double strand, and are thus larger than antisense miRNAs, leading to further difficulty with cell membrane permeability. Therefore, miRNA mimic therapy in general requires a DDS, such as lipid nanoparticles, polyplexes, and polymeric micelles [166]. Exosome may be the natural DDS to transfer anti-miR or miR-mimics. There are two approaches indicated, post-loading and pre-loading, both of which still have difficulties in the efficient incorporation of target nucleic acids [167]. Receptors such as GalNACs can be used as asialoglycoporotein receptors [168]. In addition, miRNA mimics need to be recognized by RISC, and thus the degree of nucleic acid modification possible is also limited. Treatment with nucleic acid drugs may also cause on-target toxicity (toxicity due to binding to target RNA) and off-target toxicity (toxicity due to binding to non-target RNA) [152]. It is notable that treatment targeting miRNAs might cause unexpected side effects [169], considering that one type of miRNA may regulate more than one hundred genes, including genes of interest.

## 5. Conclusions

In the present review article, we summarize the current knowledge about miRNAs as promising biomarkers and potential therapeutic targets for AKI. Recent accumulated evidence has revealed the vital role of miRNAs in both protective and pathogenic mechanisms under AKI, and possible strategies for their application as biomarkers for the early diagnosis, prediction of mortality, and identification of the specific pathology in AKI. The identification of etiology-specific miRNAs may uncover the disease mechanisms, leading to novel therapy for these diseases. Regarding the possible strategies for therapeutic options, there are two approaches: antisense for inhibition of pathogenic miRNA, and protective miRNA-mimic injection. Although the investigation of miRNA-targeted therapy for kidney diseases has just started and needs to confront several challenges before clinical use, including DDS and off-target effects involving non-target genes and organs, this research may begin a new era in the management of AKI through the regulation of specific miRNAs in the future.

## Data Availability

No datasets were generated or analyzed during the current study.

## References

[1] Ulger F., Pehlivanlar Kucuk M., Kucuk A.O., Ilkaya N.K., Murat N., Bilgic B., Abanoz H. (2018). Evaluation of acute kidney injury (AKI) with RIFLE, AKIN, CK, and KDIGO in critically ill trauma patients. Eur. J. Trauma Emerg. Surg..

[2] Susantitaphong P., Cruz D.N., Cerda J., Abulfaraj M., Alqahtani F., Koulouridis I., Jaber B.L., Acute Kidney Injury Advisory Group of the American Society of Nephrology (2013). World incidence of AKI: A meta-analysis. Clin. J. Am. Soc. Nephrol..

[3] Hoste E.A., Bagshaw S.M., Bellomo R., Cely C.M., Colman R., Cruz D.N., Edipidis K., Forni L.G., Gomersall C.D., Govil D. (2015). Epidemiology of acute kidney injury in critically ill patients: The multinational AKI-EPI study. Intensive Care Med..

[4] Singri N., Ahya S.N., Levin M.L. (2003). Acute renal failure. JAMA.

[5] Perazella M.A., Shirali A.C. (2020). Immune checkpoint inhibitor nephrotoxicity: What do we know and what should we do?. Kidney Int..

[6] Hanna R.M., Tran N.T., Patel S.S., Hou J., Jhaveri K.D., Parikh R., Selamet U., Ghobry L., Wassef O., Barsoum M. (2020). Thrombotic Microangiopathy and Acute Kidney Injury Induced after Intravitreal Injection of Vascular Endothelial Growth Factor Inhibitors VEGF Blockade-Related TMA after Intravitreal Use. Front. Med..

[7] Bellomo R., Ronco C., Kellum J.A., Mehta R.L., Palevsky P., Acute Dialysis Quality Initiative Workgroup (2004). Acute renal failure—Definition, outcome measures, animal models, fluid therapy and information technology needs: The Second International Consensus Conference of the Acute Dialysis Quality Initiative (ADQI) Group. Crit. Care.

[8] Bhosale S.J., Kulkarni A.P. (2020). Biomarkers in Acute Kidney Injury. Indian J. Crit. Care Med..

[9] Alge J.L., Arthur J.M. (2015). Biomarkers of AKI: A review of mechanistic relevance and potential therapeutic implications. Clin. J. Am. Soc. Nephrol..

[10] Cerda J., Lameire N., Eggers P., Pannu N., Uchino S., Wang H., Bagga A., Levin A. (2008). Epidemiology of acute kidney injury. Clin. J. Am. Soc. Nephrol..

[11] Coelho S., Cabral G., Lopes J.A., Jacinto A. (2018). Renal regeneration after acute kidney injury. Nephrology.

[12] Poulsom R., Forbes S.J., Hodivala-Dilke K., Ryan E., Wyles S., Navaratnarasah S., Jeffery R., Hunt T., Alison M., Cook T. (2001). Bone marrow contributes to renal parenchymal turnover and regeneration. J. Pathol..

[13] Tsuji K., Kitamura S., Wada J. (2018). Secretomes from Mesenchymal Stem Cells against Acute Kidney Injury: Possible Heterogeneity. Stem Cells Int..

[14] Tsuji K., Kitamura S. (2015). Trophic Factors from Tissue Stem Cells for Renal Regeneration. Stem Cells Int..

[15] Kitamura S., Yamasaki Y., Kinomura M., Sugaya T., Sugiyama H., Maeshima Y., Makino H. (2005). Establishment and characterization of renal progenitor like cells from S3 segment of nephron in rat adult kidney. FASEB J..

[16] Oliver J.A., Maarouf O., Cheema F.H., Martens T.P., Al-Awqati Q. (2004). The renal papilla is a niche for adult kidney stem cells. J. Clin. Investig..

[17] Maeshima A., Yamashita S., Nojima Y. (2003). Identification of renal progenitor-like tubular cells that participate in the regeneration processes of the kidney. J. Am. Soc. Nephrol..

[18] Lombardi D., Becherucci F., Romagnani P. (2016). How much can the tubule regenerate and who does it? An open question. Nephrol. Dial. Transplant..

[19] Berger K., Bangen J.M., Hammerich L., Liedtke C., Floege J., Smeets B., Moeller M.J. (2014). Origin of regenerating tubular cells after acute kidney injury. Proc. Natl. Acad. Sci. USA.

[20] O’Brien J., Hayder H., Zayed Y., Peng C. (2018). Overview of MicroRNA Biogenesis, Mechanisms of Actions, and Circulation. Front. Endocrinol..

[21] Lee R.C., Feinbaum R.L., Ambros V. (1993). The C. elegans heterochronic gene lin-4 encodes small RNAs with antisense complementarity to lin-14. Cell.

[22] Pasquinelli A.E., Reinhart B.J., Slack F., Martindale M.Q., Kuroda M.I., Maller B., Hayward D.C., Ball E.E., Degnan B., Muller P. (2000). Conservation of the sequence and temporal expression of let-7 heterochronic regulatory RNA. Nature.

[23] Xu T., Li L.X., Jia Y., Wu Q., Zhu W., Xu Z., Zheng B., Lu X. (2022). One microRNA has the potential to target whole viral mRNAs in a given human coronavirus. Front. Microbiol..

[24] Friedman R.C., Farh K.K., Burge C.B., Bartel D.P. (2009). Most mammalian mRNAs are conserved targets of microRNAs. Genome Res..

[25] Naeli P., Winter T., Hackett A.P., Alboushi L., Jafarnejad S.M. (2023). The intricate balance between microRNA-induced mRNA decay and translational repression. FEBS J..

[26] Lu J., Clark A.G. (2012). Impact of microRNA regulation on variation in human gene expression. Genome Res..

[27] Mahtal N., Lenoir O., Tinel C., Anglicheau D., Tharaux P.L. (2022). MicroRNAs in kidney injury and disease. Nat. Rev. Nephrol..

[28] Wu Y.L., Li H.F., Chen H.H., Lin H. (2020). MicroRNAs as Biomarkers and Therapeutic Targets in Inflammation- and Ischemia-Reperfusion-Related Acute Renal Injury. Int. J. Mol. Sci..

[29] Suarez B., Sole C., Marquez M., Nanetti F., Lawrie C.H. (2022). Circulating MicroRNAs as Cancer Biomarkers in Liquid Biopsies. Adv. Exp. Med. Biol..

[30] Connor K.L., Denby L. (2021). MicroRNAs as non-invasive biomarkers of renal disease. Nephrol. Dial. Transplant..

[31] Waikar S.S., Bonventre J.V. (2009). Creatinine kinetics and the definition of acute kidney injury. J. Am. Soc. Nephrol..

[32] Jana S., Mitra P., Roy S. (2022). Proficient Novel Biomarkers Guide Early Detection of Acute Kidney Injury: A Review. Diseases.

[33] Zdziechowska M., Gluba-Brzozka A., Poliwczak A.R., Franczyk B., Kidawa M., Zielinska M., Rysz J. (2020). Serum NGAL, KIM-1, IL-18, L-FABP: New biomarkers in the diagnostics of acute kidney injury (AKI) following invasive cardiology procedures. Int. Urol. Nephrol..

[34] Zou C., Wang C., Lu L. (2022). Advances in the study of subclinical AKI biomarkers. Front. Physiol..

[35] Rosenqvist M., Bronton K., Hartmann O., Bergmann A., Struck J., Melander O. (2019). Proenkephalin a 119-159 (penKid)—A novel biomarker for acute kidney injury in sepsis: An observational study. BMC Emerg. Med..

[36] Breglia A., Godi I., Virzi G.M., Guglielmetti G., Iannucci G., De Cal M., Brocca A., Carta M., Giavarina D., Ankawi G. (2020). Subclinical Contrast-Induced Acute Kidney Injury in Patients Undergoing Cerebral Computed Tomography. Cardiorenal Med..

[37] Pavkovic M., Vaidya V.S. (2016). MicroRNAs and drug-induced kidney injury. Pharmacol. Ther..

[38] Keller A., Groger L., Tschernig T., Solomon J., Laham O., Schaum N., Wagner V., Kern F., Schmartz G.P., Li Y. (2022). miRNATissueAtlas2: An update to the human miRNA tissue atlas. Nucleic Acids Res..

[39] Mitchell P.S., Parkin R.K., Kroh E.M., Fritz B.R., Wyman S.K., Pogosova-Agadjanyan E.L., Peterson A., Noteboom J., O’Briant K.C., Allen A. (2008). Circulating microRNAs as stable blood-based markers for cancer detection. Proc. Natl. Acad. Sci. USA.

[40] Aguado-Fraile E., Ramos E., Conde E., Rodriguez M., Martin-Gomez L., Lietor A., Candela A., Ponte B., Liano F., Garcia-Bermejo M.L. (2015). A Pilot Study Identifying a Set of microRNAs As Precise Diagnostic Biomarkers of Acute Kidney Injury. PLoS ONE.

[41] Chen H.H., Lan Y.F., Li H.F., Cheng C.F., Lai P.F., Li W.H., Lin H. (2016). Urinary miR-16 transactivated by C/EBPbeta reduces kidney function after ischemia/reperfusion-induced injury. Sci. Rep..

[42] Lorenzen J.M., Kielstein J.T., Hafer C., Gupta S.K., Kumpers P., Faulhaber-Walter R., Haller H., Fliser D., Thum T. (2011). Circulating miR-210 predicts survival in critically ill patients with acute kidney injury. Clin. J. Am. Soc. Nephrol..

[43] Lan Y.F., Chen H.H., Lai P.F., Cheng C.F., Huang Y.T., Lee Y.C., Chen T.W., Lin H. (2012). MicroRNA-494 reduces ATF3 expression and promotes AKI. J. Am. Soc. Nephrol..

[44] Ramachandran K., Saikumar J., Bijol V., Koyner J.L., Qian J., Betensky R.A., Waikar S.S., Vaidya V.S. (2013). Human miRNome profiling identifies microRNAs differentially present in the urine after kidney injury. Clin. Chem..

[45] Aomatsu A., Kaneko S., Yanai K., Ishii H., Ito K., Hirai K., Ookawara S., Kobayashi Y., Sanui M., Morishita Y. (2022). MicroRNA expression profiling in acute kidney injury. Transl. Res..

[46] Saikumar J., Hoffmann D., Kim T.M., Gonzalez V.R., Zhang Q., Goering P.L., Brown R.P., Bijol V., Park P.J., Waikar S.S. (2012). Expression, circulation, and excretion profile of microRNA-21, -155, and -18a following acute kidney injury. Toxicol. Sci..

[47] Gutierrez-Escolano A., Santacruz-Vazquez E., Gomez-Perez F. (2015). Dysregulated microRNAs involved in contrast-induced acute kidney injury in rat and human. Ren. Fail..

[48] Sun S.Q., Zhang T., Ding D., Zhang W.F., Wang X.L., Sun Z., Hu L.H., Qin S.Y., Shen L.H., He B. (2016). Circulating MicroRNA-188, -30a, and -30e as Early Biomarkers for Contrast-Induced Acute Kidney Injury. J. Am. Heart Assoc..

[49] Huo R., Dai M., Fan Y., Zhou J.Z., Li L., Zu J. (2017). Predictive value of miRNA-29a and miRNA-10a-5p for 28-day mortality in patients with sepsis-induced acute kidney injury. Nan Fang Yi Ke Da Xue Xue Bao.

[50] Zhang J., Wang C.J., Tang X.M., Wei Y.K. (2018). Urinary miR-26b as a potential biomarker for patients with sepsis-associated acute kidney injury: A Chinese population-based study. Eur. Rev. Med. Pharmacol. Sci..

[51] Lin Y., Ding Y., Song S., Li M., Wang T., Guo F. (2019). Expression patterns and prognostic value of miR-210, miR-494, and miR-205 in middle-aged and old patients with sepsis-induced acute kidney injury. Bosn. J. Basic. Med. Sci..

[52] Wu S.Y., Zhang H., Wu W., Wu Y.Y. (2020). Value of serum miR-21-3p in predicting acute kidney injury in children with sepsis. Zhongguo Dang Dai Er Ke Za Zhi.

[53] Zhang H., Che L., Wang Y., Zhou H., Gong H., Man X., Zhao Q. (2021). Deregulated microRNA-22-3p in patients with sepsis-induced acute kidney injury serves as a new biomarker to predict disease occurrence and 28-day survival outcomes. Int. Urol. Nephrol..

[54] Liu Z., Yang D., Gao J., Xiang X., Hu X., Li S., Wu W., Cai J., Tang C., Zhang D. (2020). Discovery and validation of miR-452 as an effective biomarker for acute kidney injury in sepsis. Theranostics.

[55] Zou Y.F., Wen D., Zhao Q., Shen P.Y., Shi H., Zhao Q., Chen Y.X., Zhang W. (2017). Urinary MicroRNA-30c-5p and MicroRNA-192-5p as potential biomarkers of ischemia-reperfusion-induced kidney injury. Exp. Biol. Med..

[56] Zhang L., Xu Y., Xue S., Wang X., Dai H., Qian J., Ni Z., Yan Y. (2017). Implications of dynamic changes in miR-192 expression in ischemic acute kidney injury. Int. Urol. Nephrol..

[57] Kang Z., Li Z., Huang P., Luo J., Liu P., Wang Y., Xia T., Zhou Y. (2018). Remote ischemic preconditioning upregulates microRNA-21 to protect the kidney in children with congenital heart disease undergoing cardiopulmonary bypass. Pediatr. Nephrol..

[58] Du J., Cao X., Zou L., Chen Y., Guo J., Chen Z., Hu S., Zheng Z. (2013). MicroRNA-21 and risk of severe acute kidney injury and poor outcomes after adult cardiac surgery. PLoS ONE.

[59] Wu R., Wu Y., Yang L., Deng Y., Chen D. (2019). Value of serum level of microRNA-494 in predicting prognosis of acute renal injury after cardiac surgery in children. Zhonghua Wei Zhong Bing Ji Jiu Yi Xue.

[60] Arvin P., Samimagham H.R., Montazerghaem H., Khayatian M., Mahboobi H., Ghadiri Soufi F. (2017). Early detection of cardiac surgery-associated acute kidney injury by microRNA-21. Bratisl. Lek. Listy.

[61] Gaede L., Liebetrau C., Blumenstein J., Troidl C., Dorr O., Kim W.K., Gottfried K., Voss S., Berkowitsch A., Walther T. (2016). Plasma microRNA-21 for the early prediction of acute kidney injury in patients undergoing major cardiac surgery. Nephrol. Dial. Transplant..

[62] Mousavi M.Z., Chen H.Y., Lee K.L., Lin H., Chen H.H., Lin Y.F., Wong C.S., Li H.F., Wei P.K., Cheng J.Y. (2015). Urinary micro-RNA biomarker detection using capped gold nanoslit SPR in a microfluidic chip. Analyst.

[63] Khwaja A. (2012). KDIGO clinical practice guidelines for acute kidney injury. Nephron Clin. Pract..

[64] Tsuji K., Kitamura S., Sang Y., Fukushima K., Wada J. (2020). Adult kidney stem/progenitor cells contribute to regeneration through the secretion of trophic factors. Stem Cell Res..

[65] Toyohara T., Mae S., Sueta S., Inoue T., Yamagishi Y., Kawamoto T., Kasahara T., Hoshina A., Toyoda T., Tanaka H. (2015). Cell Therapy Using Human Induced Pluripotent Stem Cell-Derived Renal Progenitors Ameliorates Acute Kidney Injury in Mice. Stem Cells Transl. Med..

[66] Kinomura M., Kitamura S., Tanabe K., Ichinose K., Hirokoshi K., Takazawa Y., Kitayama H., Nasu T., Sugiyama H., Yamasaki Y. (2008). Amelioration of cisplatin-induced acute renal injury by renal progenitor-like cells derived from the adult rat kidney. Cell Transplant..

[67] Tsuji K., Kitamura S., Wada J. (2020). Immunomodulatory and Regenerative Effects of Mesenchymal Stem Cell-Derived Extracellular Vesicles in Renal Diseases. Int. J. Mol. Sci..

[68] Collino F., Bruno S., Incarnato D., Dettori D., Neri F., Provero P., Pomatto M., Oliviero S., Tetta C., Quesenberry P.J. (2015). AKI Recovery Induced by Mesenchymal Stromal Cell-Derived Extracellular Vesicles Carrying MicroRNAs. J. Am. Soc. Nephrol..

[69] Mima A. (2021). Hypoxia-inducible factor-prolyl hydroxylase inhibitors for renal anemia in chronic kidney disease: Advantages and disadvantages. Eur. J. Pharmacol..

[70] Shu S., Wang Y., Zheng M., Liu Z., Cai J., Tang C., Dong Z. (2019). Hypoxia and Hypoxia-Inducible Factors in Kidney Injury and Repair. Cells.

[71] Song N., Zhang T., Xu X., Lu Z., Yu X., Fang Y., Hu J., Jia P., Teng J., Ding X. (2018). miR-21 Protects Against Ischemia/Reperfusion-Induced Acute Kidney Injury by Preventing Epithelial Cell Apoptosis and Inhibiting Dendritic Cell Maturation. Front. Physiol..

[72] Wei Q., Sun H., Song S., Liu Y., Liu P., Livingston M.J., Wang J., Liang M., Mi Q.S., Huo Y. (2018). MicroRNA-668 represses MTP18 to preserve mitochondrial dynamics in ischemic acute kidney injury. J. Clin. Investig..

[73] Wei Q., Liu Y., Liu P., Hao J., Liang M., Mi Q.S., Chen J.K., Dong Z. (2016). MicroRNA-489 Induction by Hypoxia-Inducible Factor-1 Protects against Ischemic Kidney Injury. J. Am. Soc. Nephrol..

[74] Wei Q., Bhatt K., He H.Z., Mi Q.S., Haase V.H., Dong Z. (2010). Targeted deletion of Dicer from proximal tubules protects against renal ischemia-reperfusion injury. J. Am. Soc. Nephrol..

[75] Gu D., Zou X., Ju G., Zhang G., Bao E., Zhu Y. (2016). Mesenchymal Stromal Cells Derived Extracellular Vesicles Ameliorate Acute Renal Ischemia Reperfusion Injury by Inhibition of Mitochondrial Fission through miR-30. Stem Cells Int..

[76] Zhang C., Yu S., Zheng B., Liu D., Wan F., Ma Y., Wang J., Gao Z., Shan Z. (2019). miR-30c-5p Reduces Renal Ischemia-Reperfusion Involving Macrophage. Med. Sci. Monit..

[77] Zhang Y., Li C., Guan C., Zhou B., Wang L., Yang C., Zhen L., Dai J., Zhao L., Jiang W. (2020). MiR-181d-5p Targets KLF6 to Improve Ischemia/Reperfusion-Induced AKI Through Effects on Renal Function, Apoptosis, and Inflammation. Front. Physiol..

[78] Huang S.J., Huang J., Yan Y.B., Qiu J., Tan R.Q., Liu Y., Tian Q., Guan L., Niu S.S., Zhang Y. (2020). The renoprotective effect of curcumin against cisplatin-induced acute kidney injury in mice: Involvement of miR-181a/PTEN axis. Ren. Fail..

[79] Yi H.X., Jiang S.Y., Yu L.H., Chen K., Yang Z.X., Wu Q. (2021). MicroRNA 181a-2-3p Alleviates the Apoptosis of Renal Tubular Epithelial Cells via Targeting GJB2 in Sepsis-Induced Acute Kidney Injury. Mol. Cell Biol..

[80] Zhang M., Zhi D., Lin J., Liu P., Wang Y., Duan M. (2022). miR-181a-5p Inhibits Pyroptosis in Sepsis-Induced Acute Kidney Injury through Downregulation of NEK7. J. Immunol. Res..

[81] Zhang P., Yi L., Qu S., Dai J., Li X., Liu B., Li H., Ai K., Zheng P., Qiu S. (2020). The Biomarker TCONS_00016233 Drives Septic AKI by Targeting the miR-22-3p/AIFM1 Signaling Axis. Mol. Ther. Nucleic Acids.

[82] Zhang J., Chen Q., Dai Z., Pan H. (2023). miR-22 alleviates sepsis-induced acute kidney injury via targeting the HMGB1/TLR4/NF-kappaB signaling pathway. Int. Urol. Nephrol..

[83] Ma J., Li Y.T., Zhang S.X., Fu S.Z., Ye X.Z. (2019). MiR-590-3p Attenuates Acute Kidney Injury by Inhibiting Tumor Necrosis Factor Receptor-Associated Factor 6 in Septic Mice. Inflammation.

[84] Chen Y., Zhang C., Du Y., Yang X., Liu M., Yang W., Lei G., Wang G. (2022). Exosomal transfer of microRNA-590-3p between renal tubular epithelial cells after renal ischemia-reperfusion injury regulates autophagy by targeting TRAF6. Chin. Med. J..

[85] Ding C., Dou M., Wang Y., Li Y., Wang Y., Zheng J., Li X., Xue W., Ding X., Tian P. (2020). miR-124/IRE-1alpha affects renal ischemia/reperfusion injury by regulating endoplasmic reticulum stress in renal tubular epithelial cells. Acta Biochim. Biophys. Sin..

[86] Ke J., Zhao F., Luo Y., Deng F., Wu X. (2021). MiR-124 Negatively Regulated PARP1 to Alleviate Renal Ischemia-reperfusion Injury by Inhibiting TNFalpha/RIP1/RIP3 Pathway. Int. J. Biol. Sci..

[87] Hao J., Wei Q., Mei S., Li L., Su Y., Mei C., Dong Z. (2017). Induction of microRNA-17-5p by p53 protects against renal ischemia-reperfusion injury by targeting death receptor 6. Kidney Int..

[88] Chen S., Yao Y., Lin F., Bian F., Zhu C., Jiang G. (2019). MiR-424 is over-expressed and attenuates ischemia-reperfusion kidney injury via p53 and death receptor 6 pathway. Am. J. Transl. Res..

[89] Vinas J.L., Burger D., Zimpelmann J., Haneef R., Knoll W., Campbell P., Gutsol A., Carter A., Allan D.S., Burns K.D. (2016). Transfer of microRNA-486-5p from human endothelial colony forming cell-derived exosomes reduces ischemic kidney injury. Kidney Int..

[90] Qin Y., Wang G., Peng Z. (2019). MicroRNA-191-5p diminished sepsis-induced acute kidney injury through targeting oxidative stress responsive 1 in rat models. Biosci. Rep..

[91] Zheng G., Qu H., Li F., Ma W., Yang H. (2018). Propofol attenuates sepsis-induced acute kidney injury by regulating miR-290-5p/CCL-2 signaling pathway. Braz. J. Med. Biol. Res..

[92] Li X.Y., Zhang K., Jiang Z.Y., Cai L.H. (2014). MiR-204/miR-211 downregulation contributes to candidemia-induced kidney injuries via derepression of Hmx1 expression. Life Sci..

[93] Xu Y., Jiang W., Zhong L., Li H., Bai L., Chen X., Lin Y., Zheng D. (2020). miR-195-5p alleviates acute kidney injury through repression of inflammation and oxidative stress by targeting vascular endothelial growth factor A. Aging.

[94] He X., Wen Y., Wang Q., Wang Y., Zhang G., Wu J., Li Z., Wen J. (2021). Apigenin Nanoparticle Attenuates Renal Ischemia/Reperfusion Inflammatory Injury by Regulation of miR-140-5p/CXCL12/NF-kappaB Signaling Pathway. J. Biomed. Nanotechnol..

[95] Liao W., Fu Z., Zou Y., Wen D., Ma H., Zhou F., Chen Y., Zhang M., Zhang W. (2017). Corrigendum to “MicroRNA-140-5p attenuated oxidative stress in Cisplatin induced acute kidney injury by activating Nrf2/ARE pathway through a Keap1-independent mechanism” [Exp. Cell Res. (2017) 292–302]. Exp. Cell Res..

[96] Wang Y., Wang D., Jin Z. (2019). miR-27a suppresses TLR4-induced renal ischemia-reperfusion injury. Mol. Med. Rep..

[97] Tian X., Ji Y., Liang Y., Zhang J., Guan L., Wang C. (2019). LINC00520 targeting miR-27b-3p regulates OSMR expression level to promote acute kidney injury development through the PI3K/AKT signaling pathway. J. Cell Physiol..

[98] Li X., Liao J., Su X., Li W., Bi Z., Wang J., Su Q., Huang H., Wei Y., Gao Y. (2020). Human urine-derived stem cells protect against renal ischemia/reperfusion injury in a rat model via exosomal miR-146a-5p which targets IRAK1. Theranostics.

[99] Amrouche L., Desbuissons G., Rabant M., Sauvaget V., Nguyen C., Benon A., Barre P., Rabate C., Lebreton X., Gallazzini M. (2017). MicroRNA-146a in Human and Experimental Ischemic AKI: CXCL8-Dependent Mechanism of Action. J. Am. Soc. Nephrol..

[100] Liu F., Lou Y.L., Wu J., Ruan Q.F., Xie A., Guo F., Cui S.P., Deng Z.F., Wang Y. (2012). Upregulation of microRNA-210 regulates renal angiogenesis mediated by activation of VEGF signaling pathway under ischemia/perfusion injury in vivo and in vitro. Kidney Blood Press. Res..

[101] Bijkerk R., van Solingen C., de Boer H.C., van der Pol P., Khairoun M., de Bruin R.G., van Oeveren-Rietdijk A.M., Lievers E., Schlagwein N., van Gijlswijk D.J. (2014). Hematopoietic microRNA-126 protects against renal ischemia/reperfusion injury by promoting vascular integrity. J. Am. Soc. Nephrol..

[102] Yue J., Si Y., Zhu T., Yang J., Xu X., Fang Y., Fu W. (2019). MicroRNA-187 Reduces Acute Ischemic Renal Podocyte Injury via Targeting Acetylcholinesterase. J. Surg. Res..

[103] Zhu G., Pei L., Lin F., Yin H., Li X., He W., Liu N., Gou X. (2019). Exosomes from human-bone-marrow-derived mesenchymal stem cells protect against renal ischemia/reperfusion injury via transferring miR-199a-3p. J. Cell Physiol..

[104] Yuan X., Wang X., Chen C., Zhou J., Han M. (2017). Bone mesenchymal stem cells ameliorate ischemia/reperfusion-induced damage in renal epithelial cells via microRNA-223. Stem Cell Res. Ther..

[105] Wu L., Rong C., Zhou Q., Zhao X., Zhuansun X.M., Wan S., Sun M.M., Wang S.L. (2020). Bone Marrow Mesenchymal Stem Cells Ameliorate Cisplatin-Induced Renal Fibrosis via miR-146a-5p/Tfdp2 Axis in Renal Tubular Epithelial Cells. Front. Immunol..

[106] Wilflingseder J., Jelencsics K., Bergmeister H., Sunzenauer J., Regele H., Eskandary F., Reindl-Schwaighofer R., Kainz A., Oberbauer R. (2017). miR-182-5p Inhibition Ameliorates Ischemic Acute Kidney Injury. Am. J. Pathol..

[107] Li H., Ma Y., Chen B., Shi J. (2018). miR-182 enhances acute kidney injury by promoting apoptosis involving the targeting and regulation of TCF7L2/Wnt/beta-catenins pathway. Eur. J. Pharmacol..

[108] Du Y., Ning J.Z. (2021). MiR-182 Promotes Ischemia/Reperfusion-Induced Acute Kidney Injury in Rat by Targeting FoxO3. Urol. Int..

[109] Lee C.G., Kim J.G., Kim H.J., Kwon H.K., Cho I.J., Choi D.W., Lee W.H., Kim W.D., Hwang S.J., Choi S. (2014). Discovery of an integrative network of microRNAs and transcriptomics changes for acute kidney injury. Kidney Int..

[110] Wang J., Li H., Qiu S., Dong Z., Xiang X., Zhang D. (2017). MBD2 upregulates miR-301a-5p to induce kidney cell apoptosis during vancomycin-induced AKI. Cell Death Dis..

[111] Hao J., Lou Q., Wei Q., Mei S., Li L., Wu G., Mi Q.S., Mei C., Dong Z. (2017). MicroRNA-375 Is Induced in Cisplatin Nephrotoxicity to Repress Hepatocyte Nuclear Factor 1-beta. J. Biol. Chem..

[112] Liu B., Chai Y., Guo W., Lin K., Chen S., Liu J., Sun G., Chen G., Song F., He Y. (2019). MicroRNA-188 aggravates contrast-induced apoptosis by targeting SRSF7 in novel isotonic contrast-induced acute kidney injury rat models and renal tubular epithelial cells. Ann. Transl. Med..

[113] Liu X., Zhu N., Zhang B., Xu S.B. (2020). Long Noncoding RNA TCONS_00016406 Attenuates Lipopolysaccharide-Induced Acute Kidney Injury by Regulating the miR-687/PTEN Pathway. Front. Physiol..

[114] Lorenzen J.M., Kaucsar T., Schauerte C., Schmitt R., Rong S., Hubner A., Scherf K., Fiedler J., Martino F., Kumarswamy R. (2014). MicroRNA-24 antagonism prevents renal ischemia reperfusion injury. J. Am. Soc. Nephrol..

[115] Zhang T., Xiang L. (2019). Honokiol alleviates sepsis-induced acute kidney injury in mice by targeting the miR-218-5p/heme oxygenase-1 signaling pathway. Cell Mol. Biol. Lett..

[116] Chen L., Xu J.Y., Tan H.B. (2021). LncRNA TUG1 regulates the development of ischemia-reperfusion mediated acute kidney injury through miR-494-3p/E-cadherin axis. J. Inflamm..

[117] Lu P., Zhang L., Liu T., Fan J.J., Luo X., Zhu Y.T. (2022). MiR-494-mediated Effects on the NF-κB Signaling Pathway Regulate Lipopolysaccharide-Induced Acute Kidney Injury in Mice. Immunol. Investig..

[118] Shen Y., Yu J., Jing Y., Zhang J. (2019). MiR-106a aggravates sepsis-induced acute kidney injury by targeting THBS2 in mice model. Acta Cir. Bras..

[119] Chen S., Shan J., Niu W., Lin F., Liu S., Wu P., Sun L., Lu W., Jiang G. (2018). Micro RNA-155 inhibitor as a potential therapeutic strategy for the treatment of acute kidney injury (AKI): A nanomedicine perspective. RSC Adv..

[120] Zhang Z., Chen H., Zhou L., Li C., Lu G., Wang L. (2022). Macrophage-derived exosomal miRNA-155 promotes tubular injury in ischemia-induced acute kidney injury. Int. J. Mol. Med..

[121] Zhang X.B., Chen X., Li D.J., Qi G.N., Dai Y.Q., Gu J., Chen M.Q., Hu S., Liu Z.Y., Yang Z.M. (2019). Inhibition of miR-155 Ameliorates Acute Kidney Injury by Apoptosis Involving the Regulation on TCF4/Wnt/beta-Catenin Pathway. Nephron.

[122] Wang Y., Wu X.Q., Cai J.R., Ji H.X., Xu T. (2023). SIRT7 silencing by miR-152-3p confers cell apoptosis and renal functional impairment induced by renal ischaemia/reperfusion injury. Int. Urol. Nephrol..

[123] Ma P., Zhang C., Huo P., Li Y., Yang H. (2020). A novel role of the miR-152-3p/ERRFI1/STAT3 pathway modulates the apoptosis and inflammatory response after acute kidney injury. J. Biochem. Mol. Toxicol..

[124] Guo Y., Ni J., Chen S., Bai M., Lin J., Ding G., Zhang Y., Sun P., Jia Z., Huang S. (2018). MicroRNA-709 Mediates Acute Tubular Injury through Effects on Mitochondrial Function. J. Am. Soc. Nephrol..

[125] Wei W., Yao Y.Y., Bi H.Y., Zhai Z., Gao Y. (2020). miR-21 protects against lipopolysaccharide-stimulated acute kidney injury and apoptosis by targeting CDK6. Ann. Transl. Med..

[126] Jia P., Pan T., Xu S., Fang Y., Song N., Guo M., Liang Y., Xu X., Ding X. (2020). Depletion of miR-21 in dendritic cells aggravates renal ischemia-reperfusion injury. FASEB J..

[127] Li Z., Deng X., Kang Z., Wang Y., Xia T., Ding N., Yin Y. (2016). Elevation of miR-21, through targeting MKK3, may be involved in ischemia pretreatment protection from ischemia-reperfusion induced kidney injury. J. Nephrol..

[128] Liu X., Hong Q., Wang Z., Yu Y., Zou X., Xu L. (2015). MiR-21 inhibits autophagy by targeting Rab11a in renal ischemia/reperfusion. Exp. Cell Res..

[129] Tang C.R., Luo S.G., Lin X., Wang J., Liu Y. (2019). Silenced miR-21 inhibits renal interstitial fibrosis via targeting ERK1/2 signaling pathway in mice. Eur. Rev. Med. Pharmacol. Sci..

[130] Lin Z., Liu Z., Wang X., Qiu C., Zheng S. (2019). MiR-21-3p Plays a Crucial Role in Metabolism Alteration of Renal Tubular Epithelial Cells during Sepsis Associated Acute Kidney Injury via AKT/CDK2-FOXO1 Pathway. BioMed Res. Int..

[131] He S.Y., Wang G., Pei Y.H., Zhu H.P. (2020). miR-34b-3p protects against acute kidney injury in sepsis mice via targeting ubiquitin-like protein 4A. Kaohsiung J. Med. Sci..

[132] Bhatt K., Zhou L., Mi Q.S., Huang S., She J.X., Dong Z. (2010). MicroRNA-34a is induced via p53 during cisplatin nephrotoxicity and contributes to cell survival. Mol. Med..

[133] Jiang Z.J., Zhang M.Y., Fan Z.W., Sun W.L., Tang Y. (2019). Influence of lncRNA HOTAIR on acute kidney injury in sepsis rats through regulating miR-34a/Bcl-2 pathway. Eur. Rev. Med. Pharmacol. Sci..

[134] Liu X.J., Hong Q., Wang Z., Yu Y.Y., Zou X., Xu L.H. (2015). MicroRNA-34a Suppresses Autophagy in Tubular Epithelial Cells in Acute Kidney Injury. Am. J. Nephrol..

[135] Collier J.B., Schnellmann R.G. (2020). Extracellular signal-regulated kinase 1/2 regulates NAD metabolism during acute kidney injury through microRNA-34a-mediated NAMPT expression. Cell Mol. Life Sci..

[136] Cao J.Y., Wang B., Tang T.T., Wen Y., Li Z.L., Feng S.T., Wu M., Liu D., Yin D., Ma K.L. (2021). Exosomal miR-125b-5p deriving from mesenchymal stem cells promotes tubular repair by suppression of p53 in ischemic acute kidney injury. Theranostics.

[137] Zhao Y., Lang Y., Zhang M., Liang S., Zhu X., Liu Z. (2022). miR-125b Disrupts Mitochondrial Dynamics via Targeting Mitofusin 1 in Cisplatin-Induced Acute Kidney Injury. Kidney Dis..

[138] Shi L., Zhang Y., Xia Y., Li C., Song Z., Zhu J. (2021). MiR-150-5p protects against septic acute kidney injury via repressing the MEKK3/JNK pathway. Cell Signal.

[139] Ranganathan P., Jayakumar C., Tang Y., Park K.M., Teoh J.P., Su H., Li J., Kim I.M., Ramesh G. (2015). MicroRNA-150 deletion in mice protects kidney from myocardial infarction-induced acute kidney injury. Am. J. Physiol. Renal Physiol..

[140] Guan H., Peng R., Mao L., Fang F., Xu B., Chen M. (2020). Injured tubular epithelial cells activate fibroblasts to promote kidney fibrosis through miR-150-containing exosomes. Exp. Cell Res..

[141] Zhou X., Zhao S., Li W., Ruan Y., Yuan R., Ning J., Jiang K., Xie J., Yao X., Li H. (2021). Tubular cell-derived exosomal miR-150-5p contributes to renal fibrosis following unilateral ischemia-reperfusion injury by activating fibroblast in vitro and in vivo. Int. J. Biol. Sci..

[142] Zhu X., Li W., Li H. (2018). miR-214 ameliorates acute kidney injury via targeting DKK3 and activating of Wnt/beta-catenin signaling pathway. Biol. Res..

[143] Sang Z., Dong S., Zhang P., Wei Y. (2021). miR-214 ameliorates sepsis-induced acute kidney injury via PTEN/AKT/mTOR-regulated autophagy. Mol. Med. Rep..

[144] Yan Y., Ma Z., Zhu J., Zeng M., Liu H., Dong Z. (2020). miR-214 represses mitofusin-2 to promote renal tubular apoptosis in ischemic acute kidney injury. Am. J. Physiol. Renal Physiol..

[145] Guo C., Ye F.X., Jian Y.H., Liu C.H., Tu Z.H., Yang D.P. (2022). MicroRNA-214-5p aggravates sepsis-related acute kidney injury in mice. Drug Dev. Res..

[146] Zhou J., Xiao C., Zheng S., Wang Q., Zhu H., Zhang Y., Wang R. (2022). MicroRNA-214-3p aggravates ferroptosis by targeting GPX4 in cisplatin-induced acute kidney injury. Cell Stress. Chaperones.

[147] Zou X., Zhang G., Cheng Z., Yin D., Du T., Ju G., Miao S., Liu G., Lu M., Zhu Y. (2014). Microvesicles derived from human Wharton’s Jelly mesenchymal stromal cells ameliorate renal ischemia-reperfusion injury in rats by suppressing CX3CL1. Stem Cell Res. Ther..

[148] Sun W., Zhu Q., Yan L., Shao F. (2019). Mesenchymal stem cells alleviate acute kidney injury via miR-107-mediated regulation of ribosomal protein S19. Ann. Transl. Med..

[149] Wang S., Zhang Z., Wang J., Miao H. (2017). MiR-107 induces TNF-alpha secretion in endothelial cells causing tubular cell injury in patients with septic acute kidney injury. Biochem. Biophys. Res. Commun..

[150] Yin Q., Zhao Y.J., Ni W.J., Tang T.T., Wang Y., Cao J.Y., Yin D., Wen Y., Li Z.L., Zhang Y.L. (2022). MiR-155 deficiency protects renal tubular epithelial cells from telomeric and genomic DNA damage in cisplatin-induced acute kidney injury. Theranostics.

[151] Thakur S., Sinhari A., Jain P., Jadhav H.R. (2022). A perspective on oligonucleotide therapy: Approaches to patient customization. Front. Pharmacol..

[152] Kulkarni J.A., Witzigmann D., Thomson S.B., Chen S., Leavitt B.R., Cullis P.R., van der Meel R. (2021). The current landscape of nucleic acid therapeutics. Nat. Nanotechnol..

[153] Yamakawa K., Nakano-Narusawa Y., Hashimoto N., Yokohira M., Matsuda Y. (2019). Development and Clinical Trials of Nucleic Acid Medicines for Pancreatic Cancer Treatment. Int. J. Mol. Sci..

[154] Lanford R.E., Hildebrandt-Eriksen E.S., Petri A., Persson R., Lindow M., Munk M.E., Kauppinen S., Orum H. (2010). Therapeutic silencing of microRNA-122 in primates with chronic hepatitis C virus infection. Science.

[155] Anastasiadou E., Seto A.G., Beatty X., Hermreck M., Gilles M.E., Stroopinsky D., Pinter-Brown L.C., Pestano L., Marchese C., Avigan D. (2021). Cobomarsen, an Oligonucleotide Inhibitor of miR-155, Slows DLBCL Tumor Cell Growth In Vitro and In Vivo. Clin. Cancer Res..

[156] Yoo B., Ghosh S.K., Kumar M., Moore A., Yigit M.V., Medarova Z. (2014). Design of nanodrugs for miRNA targeting in tumor cells. J. Biomed. Nanotechnol..

[157] Yigit M.V., Moore A., Medarova Z. (2012). Magnetic nanoparticles for cancer diagnosis and therapy. Pharm. Res..

[158] Ma L., Reinhardt F., Pan E., Soutschek J., Bhat B., Marcusson E.G., Teruya-Feldstein J., Bell G.W., Weinberg R.A. (2010). Therapeutic silencing of miR-10b inhibits metastasis in a mouse mammary tumor model. Nat. Biotechnol..

[159] Weissleder R., Lee A.S., Fischman A.J., Reimer P., Shen T., Wilkinson R., Callahan R.J., Brady T.J. (1991). Polyclonal human immunoglobulin G labeled with polymeric iron oxide: Antibody MR imaging. Radiology.

[160] Hong D.S., Kang Y.K., Borad M., Sachdev J., Ejadi S., Lim H.Y., Brenner A.J., Park K., Lee J.L., Kim T.Y. (2020). Phase 1 study of MRX34, a liposomal miR-34a mimic, in patients with advanced solid tumours. Br. J. Cancer.

[161] Zhang L., Liao Y., Tang L. (2019). MicroRNA-34 family: A potential tumor suppressor and therapeutic candidate in cancer. J. Exp. Clin. Cancer Res..

[162] Beg M.S., Brenner A.J., Sachdev J., Borad M., Kang Y.K., Stoudemire J., Smith S., Bader A.G., Kim S., Hong D.S. (2017). Phase I study of MRX34, a liposomal miR-34a mimic, administered twice weekly in patients with advanced solid tumors. Investig. New Drugs.

[163] van Zandwijk N., Pavlakis N., Kao S.C., Linton A., Boyer M.J., Clarke S., Huynh Y., Chrzanowska A., Fulham M.J., Bailey D.L. (2017). Safety and activity of microRNA-loaded minicells in patients with recurrent malignant pleural mesothelioma: A first-in-man, phase 1, open-label, dose-escalation study. Lancet Oncol..

[164] Kashtan C. (2021). Multidisciplinary Management of Alport Syndrome: Current Perspectives. J. Multidiscip. Healthc..

[165] Lee E.C., Valencia T., Allerson C., Schairer A., Flaten A., Yheskel M., Kersjes K., Li J., Gatto S., Takhar M. (2019). Discovery and preclinical evaluation of anti-miR-17 oligonucleotide RGLS4326 for the treatment of polycystic kidney disease. Nat. Commun..

[166] Meng W., He C., Hao Y., Wang L., Li L., Zhu G. (2020). Prospects and challenges of extracellular vesicle-based drug delivery system: Considering cell source. Drug Deliv..

[167] Tsuji K., Kitamura S., Wada J. (2022). Mesenchymal stem cells-derived extracellular vesicles as ‘natural’ drug delivery system for tissue regeneration. Biocell.

[168] Debacker A.J., Voutila J., Catley M., Blakey D., Habib N. (2020). Delivery of Oligonucleotides to the Liver with GalNAc: From Research to Registered Therapeutic Drug. Mol. Ther..

[169] Kim T., Croce C.M. (2023). MicroRNA: Trends in clinical trials of cancer diagnosis and therapy strategies. Exp. Mol. Med..

